# Impact of spinal sagittal malalignment on locomotive syndrome and physical function in community-dwelling middle aged and older women

**DOI:** 10.1186/s12891-023-06686-2

**Published:** 2023-07-31

**Authors:** Mio Yahata, Kei Watanabe, Hideki Tashi, Masayuki Ohashi, Takuya Yoda, Atsushi Nawata, Kazutoshi Nakamura, Hiroyuki Kawashima

**Affiliations:** 1 Division of Orthopedic Surgery, Department of Regenerative and Transplant Medicine, Niigata University Graduate School of Medical and Dental Sciences, 1-757 Asahimachi Dori, Chuo-Ku, Niigata City, Niigata 951-8510 Japan; 2 Division of Musculoskeletal Science for Frailty, Niigata University Graduate School of Medical and Dental Sciences, Niigata, Japan; 3 Division of Comprehensive Musculoskeletal Medicine, Niigata University Graduate School of Medical and Dental Sciences, Niigata, Japan; 4 Division of Preventive Medicine, Niigata University Graduate School of Medical and Dental Sciences, Niigata, Japan

**Keywords:** Adult spinal deformity, Sagittal alignment, Locomotive syndrome, Low back pain, Quality of life

## Abstract

**Background:**

Adult spinal deformity has a substantially debilitating effect on older people’s physical and mental health. However, the impact of sagittal malalignment on locomotive syndrome (LS), sarcopenia, and physical function in community-dwelling older women has not yet been clarified. This study aimed to investigate the association between these factors in community-dwelling middle aged and older women.

**Methods:**

A total of 361 women were recruited from participants performing aquatic exercises in a rural area of Japan. The body mass index, skeletal muscle mass index, trunk muscle mass, spinal inclination angle (SIA), grip strength, timed up-and-go test (TUG), maximum stride of the participants, and one-leg standing time were measured. Low back pain (LBP)- and health-related quality of life (HRQOL) were evaluated using the Oswestry Disability Index (ODI) and the Short-Form 8 questionnaire. Associations between the global sagittal alignment using SIA and investigating parameters were analyzed.

**Results:**

The prevalence of sarcopenia was 3.6%. The prevalence of LS (stages 1, 2, and 3) was 43.8% (158 of 361), and the number of participants in each LS stage was 203 (stage 0), 95 (stage 1), 28 (stage 2), and 35 (stage 3). The SIA was significantly correlated with the 25-question geriatric locomotive function scale (*r*′ = 0.292, *p* < 0.001), ODI (*r*′ = 0.267, *p* < 0.001), and TUG (*r*′ = 0.453, *p* < 0.001) after adjusting for age. In the receiver-operating characteristic curve analysis, the cutoff values of SIA for LS ≥ stage 2 and ODI ≥ 20% were 5°.

**Conclusions:**

LBP-related QOL and physical performance were significantly associated with global sagittal alignment. Global sagittal alignment was correlated with the three-stage category of LS. The spinal inclination of 5° was a cutoff value to predict exacerbation of mobility function and HRQOL status.

## Background

Recently, adult spinal deformity (ASD) with global sagittal malalignment and its correlation with low back pain (LBP) and health-related quality of life (HRQOL) status has received considerable attention [1, 2]. Regarding the health burden, patients with ASD, regardless of surgical and non-surgical candidates, showed worse HRQOL status than patients with any of the four chronic conditions, including self-reported arthritis, chronic lung disease, diabetes mellitus, and congestive heart failure [3]. Another study reported that sagittal deformity (sagittal vertical axis > 10 cm) demonstrated the most impact on physical function among various types of spinal deformity, which showed similar or worse disability with chronic lung disease and limited vision or function of the arms and legs [4]. Since the prevalence of the pathology has increased secondary to an aging Japanese population, further research is needed to understand the clinical impact of the disease, the risk factors of deformity progression, and the expected improvement with treatment. However, fewer studies have focused on the risk factors of deformity development or the significance of spinal sagittal malalignment on HRQOL, walking ability, and activities of daily living (ADL) in community-dwelling middle aged and older people [5–7].

As Japan enters into a super-aging society, the Japanese Orthopaedic Association (JOA) has advocated the concept of locomotive syndrome (LS) as a condition of reduced mobility function since 2007 [8]. LS is diagnosed by measuring the two-step test, stand-up test, and 25-question geriatric locomotive function scale (GLFS-25). If the scores of any of these tests fall below the respective cutoff values, the participant is diagnosed with LS [9]. In the 2015 proposals, LS was classified into two stages (LS stage 1, the beginning of a decline in mobility function; stage 2, progression of a decline in mobility function). In a 2020 proposal, the JOA added the criteria of LS stage 3, which indicates a progressive decline in mobility function and hindered social participation similar to that of physical frailty [10].

Sarcopenia is defined as a disease in which skeletal muscle strength interferes with daily life [11]. Skeletal muscle mass decreases linearly in both upper and lower limbs after 50 years of age [12, 13], and sarcopenia is most common in older people. Sarcopenia is caused by physical disabilities and several diseases, such as diabetes mellitus and osteoporosis [14, 15]. Sarcopenia is diagnosed by measuring the skeletal muscle mass index (SMI), walking speed and grip strength, respectively [16]. In the Asian Working Group for Sarcopenia (AWGS) 2014 proposals, if the walking speed and/or grip strength fall below the respective cutoff values, in adittion, if a decrease in skeletal muscle mass below the cutoff value is observed, the participant is diagnosed with sarcopenia [17]. In the AWGS 2019 proposals, short physical performance battery score and 5-time chair stand test were added to physical performance test before final skeletal muscle testing [18].

Since chronic LBP associated with sagittal malalignment is one of the most frequent symptoms, precise pathologic analysis should be conducted to establish preventive measures against this condition and extend healthy life expectancy. However, the relationships between global sagittal alignment and physical conditions, including LS and sarcopenia, among middle aged and older people have not been fully investigated. The present study aimed to investigate the association between these factors among middle aged and older women through a cross-sectional community-dwelling study.

## Methods

### Participants and study design

We held a locomotor examination for participants performing in an aquatic exercise program in Agano City, Niigata Prefecture, Japan, in 2019. From a total of 403 participants in this examination, 361 women with a mean age of 71.1 ± 6.0 years (range, 53–88 years) who completed all following investigation items were enrolled in this study. The study protocol was approved by the Ethics Committee of Niigata University (approval number 2019–0268), and all participants provided written informed consent.

### Evaluation of physical status, sarcopenia, and spinal sagittal alignment

We measured the height, body weight, trunk, and appendicular skeletal muscle mass of the participants using bioelectrical impedance with a body composition analyzer (MC-780A-N, Tanita Corp, Tokyo, Japan). Body mass index (BMI) was calculated by dividing the body weight by height in square meters (kg/m^2^), and SMI was calculated by dividing the appendicular skeletal muscle mass by height in square meters (kg/m^2^). Here, sarcopenia was defined as grip strength (GS) of < 18 kg and SMI of < 5.7 kg/m^2^ for women based on the Asian Working Group for Sarcopenia (AWGS) algorism in 2019 [18], while those with ≥ 18 kg or ≥ 5.7 kg/m^2^ were categorized as the non-sarcopenia group.

To evaluate global sagittal alignment, we used the Spinal Mouse® (Idiag, Fehraltorf, Switzerland), a non-invasive device used to measure the participants’ spinal sagittal alignment in a neutral standing position. The global sagittal alignment was assessed by the spinal inclination angle (SIA), calculated automatically by the Spinal Mouse®, from the tip of the T1 spinous process to that of S1. Positive SIA values indicated that the participant’s posture was inclined forward. In addition, the thoracic kyphosis angle (TKA) (Cobb angle between T1 and T12) and the lumbar lordosis angle (LLA) (Cobb angle between T12 and S1) were also calculated. The intraclass correlation coefficients for curvature measurement with Spinal Mouse® have been reported as 0.92–0.95 [19].

### Evaluation of physical performance

We performed four physical performance tests: GS, TUG, maximum stride, and one-leg standing time with eyes open (OLS). The GS was measured bilaterally in a standing position using a handgrip dynamometer. Both hands were tested, and the higher value was used as the measured value. The TUG was defined as the time it took a participant to stand up from a standard chair, walk a distance of 3 m, turn around at a corner, walk back to the chair, and sit down. Each participant performed the test once at a maximum pace, and the time was used for analyses. The maximum stride was measured from a standing position. The participants placed one foot forward as far as possible without touching their hands on the floor. All participants repeated the procedure bilaterally, and the average value was used as the maximum stride. The OLS was defined as the time from when the participant raised their leg until the leg was put down on the floor, or up to a maximum of 60 s. We measured the time for each leg raise and used the average value for analysis.

### Assessment of LS

We used the GLFS-25 score in the present study to assess LS [20]. Moreover, we adopted a 25-item questionnaire, which all participants answered. The adequate response rate was 83% (250 of 302 participants). Based on the GLFS-25 results, the participants were categorized into a non-LS (stage 0) and LS (three stages) group, as per the JOA definition [10]. In the LS group, the criterion for LS stage 1 was a GLFS-25 score from 7 to 15, indicating the beginning of mobility function decline; for LS stage 2, the criterion was a score from 16 to 23, which indicated a progression in mobility function decline, requiring some support in daily life, and for LS stage 3, the criterion was a score of 24 or more, indicating further progression in mobility function decline causing some difficulty in social participation.

### Assessment of LBP and HRQOL

LBP was evaluated using the Oswestry Disability Index (ODI). A higher ODI percentage (ODI%) indicated a lower quality of life due to LBP impairment, and the threshold value of the ODI ≥ 20% corresponding to moderate to severe disability was employed for the analysis [21]. HRQOL was assessed using the Short-Form 8 questionnaire (SF-8), which simplifies the Short-Form 36 questionnaire version 2 (SF-36v2) and consists of only eight items. Like the SF-36v2, the SF-8 covers eight categories: physical functioning, physical role, bodily pain, general health, vitality, social functioning, emotional role, and mental health. The scores of physical component summary (PCS) and mental component summary (MCS) were calculated from these eight domains. These questionnaires were answered before the examination and returned on the examination day.

### Statistical analysis

All continuous data were expressed as mean values (standard deviation). All statistical analyses were performed using the SPSS Version 19.0 (SPSS Inc., Chicago, IL, USA). First, the participants were classified into non-sarcopenia and sarcopenia groups based on GS and SMI, and the SIAs were compared between the two groups using the Mann–Whitney U test. Second, the participants were classified into four groups according to the stage (LS stage 0, 1, 2, and 3) based on the GLF-25, and the SIAs were compared among the four groups using the Kruskal–Wallis test and Bonferroni correction. Third, bivariable associations between the SIAs and the contributing factors were evaluated using Pearson’s correlation coefficient (*r*). Additionally, Pearson’s partial correlations (*r*′) were calculated to evaluate the associations while adjusting for age. The receiver-operating characteristic (ROC) curve was plotted to investigate the cutoff values of the SIA for target endpoints. Finally, the participants were assigned to the kyphosis and non-kyphosis groups based on the cutoff values of SIA, as mentioned above. All aforementioned investigation parameters were compared between the two groups using the Mann–Whitney U test. A correlation coefficient with an absolute value ≥ 0.25 and *p*-value < 0.05 were considered statistically significant.

## Results

### Background characteristics

The background characteristics of the 361 participants are shown in Table 1. The prevalence of sarcopenia was 3.6% (13 of 361). The prevalence of LS (stages 1, 2, and 3) was 43.8% (158 of 361), and the number of participants in each LS stage was 203 (stage 0), 95 (stage 1), 28 (stage 2), and 35 (stage 3). The prevalence of LBP (ODI ≥ 20%) was 32.7% (118 of 361).

**Table 1:** Background characteristics of the participants

Characteristics	Total (*N* = 361)
Age (year)	71.1 ± 6.0
BMI (kg/m^2^)	23.4 ± 3.4
Trunk muscle mass (kg)	19.9 ± 1.6
SMI (kg/m^2^)	6.3 ± 0.8
GLFS-25 (point)	9.1 ± 10.0
ODI (%)	15.0 ± 13.6
SF-8 PCS (point)	46.4 ± 7.6
SF-8 MCS (point)	51.2 ± 6.1
SIA (°)	4.5 ± 5.0
TKA (°)	40.9 ± 13.6
LLA (°)	17.2 ± 15.4
Grip strength (kg)	23.1 ± 4.0
TUG (second)	5.3 ± 1.1
Maximum stride (cm)	110.0 ± 20.2
OLS (second)	32.3 ± 20.8

All values are represented as the mean ± standard deviation

*Abbreviations*: *LS* locomotive syndrome, *BMI* body mass index, *SMI* skeletal muscle mass index, *GLFS-25* 25-question Geriatric Locomotive Function Scale, *ODI* Oswestry Disability Index, *SF-8* Short Form-8 questionnaire, *PCS* physical component summary, *MCS* mental component summary, *SIA* spinal inclination angle, *TKA* thoracic kyphosis angle, *LLA* lumbar lordosis angle, *TUG* timed up-and-go test, *OLS* one-leg standing time

### Correlation between SIA and the investigating parameters

**Fig. 1:**
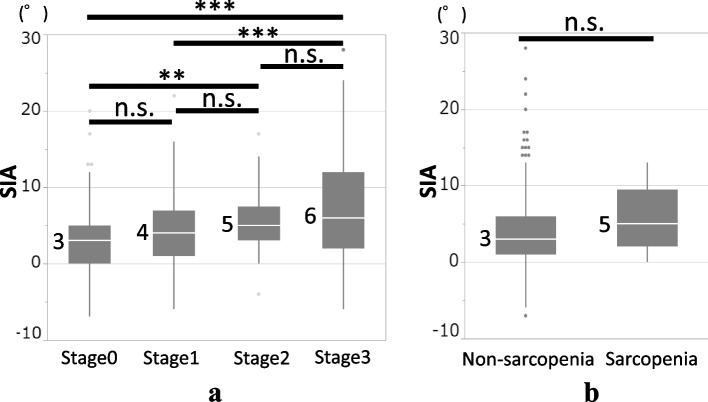
**A** Comparisons of SIA among the four groups (LS stage 0, stage 1, stage 2, and stage 3) according to the GLFS-25 score. **B** Comparisons of SIA between the two groups (non-sarcopenia and sarcopenia) according to the grip strength and skeletal muscle mass index. The number beside each box indicates the median value. *p < 0.05, **p < 0.01, ***p < 0.001. Abbreviations: SIA, spinal inclination angle; LS, locomotive syndrome; GLFS-25, 25-question geriatric locomotive function scale

The results of the correlation analysis between SIA and the investigating parameters are shown in Table 2. The SIA showed significant correlations with the GLF-25 (*r* = 0.312), ODI% (*r* = 0.284), and TUG (*r* = 0.501). After adjusting for age, which was considered a definitive contributing factor to spinal alignment, the GLF-25 (*r*′ = 0.292), ODI% (*r*′ = 0.267), and TUG (*r*′ = 0.453) remained significantly correlated with the SIA. As shown in Fig. 1, the SIA gradually deteriorated as the LS stage progressed, whereas sarcopenia did not affect spinal alignment.

**Table 2:** Correlations between SIA and the investigation items using Pearson correlation coefficient

Variables	Correlation coefficient	p-value	Age-adjusted correlation coefficient	*p*-value
Age (year)	0.247	< 0.001	-	-
BMI (kg/m^2^)	0.030	0.576	0.056	0.298
Trunk muscle mass (kg)	-0.212	< 0.001	-0.179	< 0.001
SMI (kg/m^2^)	0.044	0.401	0.099	0.063
GLFS-25 (point)	**0.312**	** < 0.001**	**0.292**	** < 0.001**
ODI (%)	**0.284**	** < 0.001**	**0.267**	** < 0.001**
SF-8 PCS (point)	-0.210	< 0.001	-0.203	< 0.001
SF-8 MCS (point)	-0.022	0.679	-0.056	0.293
TKA (°)	-**0.340**	** < 0.001**	-**0.326**	** < 0.001**
LLA (°)	**0.577**	** < 0.001**	**0.552**	** < 0.001**
Grip strength (kg)	-0.219	< 0.001	-0.162	0.002
TUG (second)	**0.501**	** < 0.001**	**0.453**	** < 0.001**
Maximum stride (cm)	-0.133	0.012	-0.147	0.006
OLS (second)	-0.172	0.001	-0.087	0.102

*Abbreviations*: *SIA* spinal inclination angle, *BMI* body mass index, *SMI* skeletal muscle mass index, *GLFS-25* 25-question Geriatric Locomotive Function Scale, *ODI* Oswestry Disability Index, *PCS* physical component summary, *MCS* mental component summary, *TKA* thoracic kyphosis angle, *LLA* lumbar lordosis angle, *TUG* timed up-and-go test, *OLS* one-leg standing time

### Impact of global sagittal alignment on physical function

**Fig. 2:**
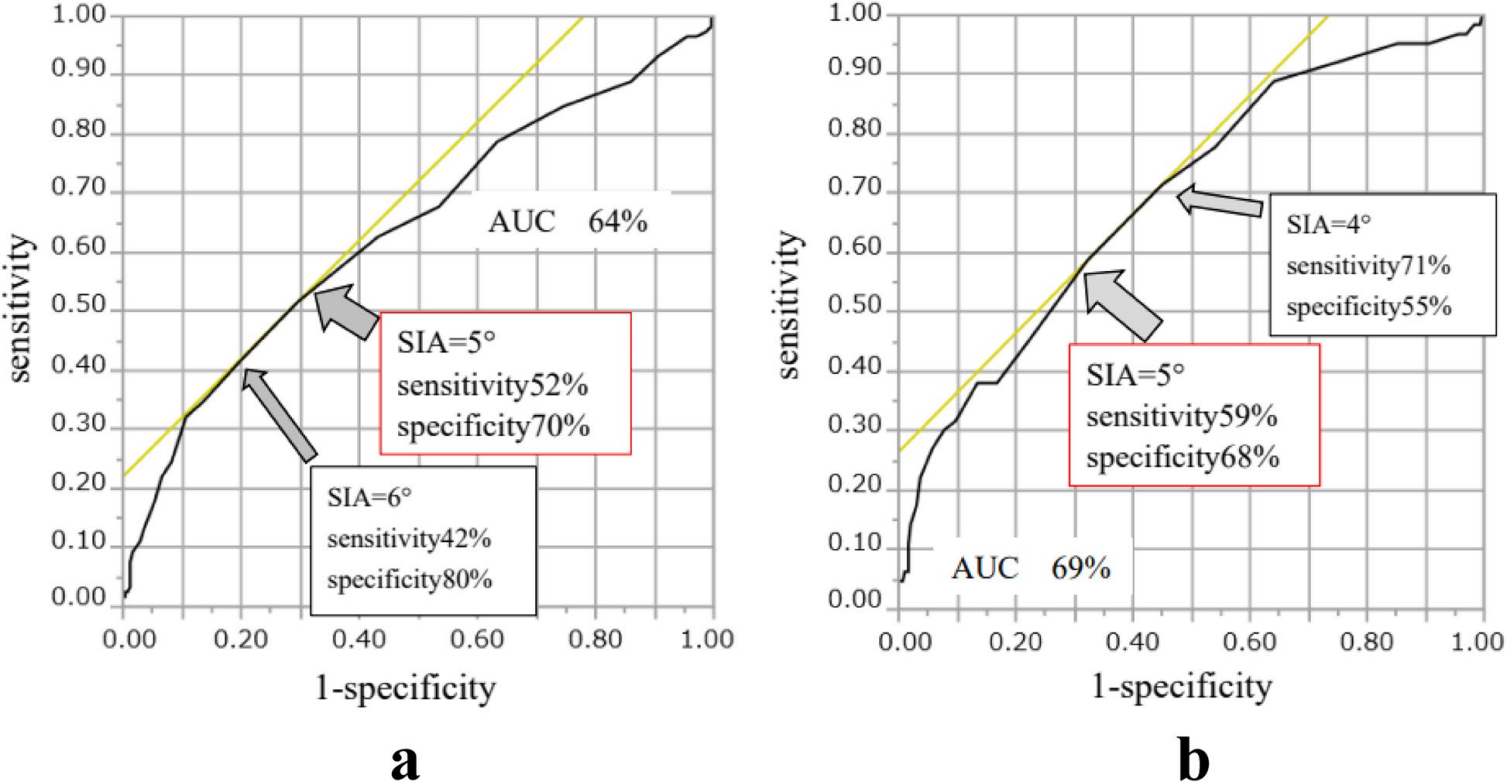
**A** ROC curves and cutoff values of the SIA corresponding to LS (a GLFS-25 score ≥ 16 points). The wider arrow and the larger font indicate the best cutoff value and corresponding sensitivity and specificity for each test group. **B** ROC curves and cutoff values of the SIA corresponding to LBP-related QOL (an ODI score ≥ 20%). The wider arrow and the larger font indicate the best cutoff value and corresponding sensitivity and specificity for each test group. Abbreviations: SIA, spinal inclination angle; LS, locomotive syndrome; GLFS-25, 25-question geriatric locomotive function scale; ROC, receiver-operating characteristic; LBP, Low back pain; QOL, quality of life; ODI, Oswestry Disability Index

In the ROC curve analysis, the cutoff values of SIA were concordantly 5° in the detection of ODI ≥ 20% (*p* < 0.0001; AUC, 0.64; sensitivity, 52%; specificity, 70%; Fig. 2a) and LS stage ≥ 2 (*p* < 0.0001; AUC, 0.69; sensitivity, 59%; specificity, 68%; Fig. 2b). In addition, the participants were divided into two groups based on the cutoff values mentioned above: a non-kyphosis group (*n* = 228) with SIA < 5° and a kyphosis group (*n* = 133) with SIA ≥ 5°. Comparing the physical function status between the two groups, participants in the kyphosis group showed lower HRQOL status using self-reported questionnaires and lower physical performance status related to skeletal muscle strength, gait ability, and trunk balance (Table 3).

**Table 3:** Comparisons of the investigation items between the non-kyphosis and kyphosis groups

Variables	Non-kyphosis group with SIA < 5° (*N* = 228)	Kyphosis group with SIA ≥ 5° (*N* = 133)	*p*-value
Age (year)	70.3 ± 5.7	72.6 ± 6.2	< 0.001
BMI (kg/m^2^)	23.4 ± 3.4	23.5 ± 3.6	0.308
Trunk muscle mass (kg)	20.0 ± 1.6	19.6 ± 1.8	0.041
SMI (kg/m^2^)	6.3 ± 0.7	6.4 ± 0.9	0.137
GLFS-25 (point)	7.6 ± 9.1	11.8 ± 10.9	< 0.001
ODI (%)	12.8 ± 12.4	18.9 ± 14.6	< 0.001
SF-8 PCS (point)	47.3 ± 7.3	45.0 ± 7.9	0.004
SF-8 MCS (point)	51.5 ± 5.7	50.7 ± 6.8	0.291
SIA (°)	1.2 ± 2.4	8.7 ± 5.0	< 0.001
TKA (°)	43.2 ± 12.7	37.1 ± 14.2	< 0.001
LLA (°)	22.3 ± 11.8	8.3 ± 16.9	< 0.001
Grip strength (kg)	23.6 ± 3.7	22.1 ± 4.4	< 0.001
TUG (second)	4.9 ± 1.2	5.6 ± 1.5	< 0.001
Maximum stride (cm)	111.1 ± 22.3	108.2 ± 16.1	< 0.001
OLS (second)	34.2 ± 20.9	29.0 ± 20.3	< 0.001

All values are represented as the mean ± standard deviation

*Abbreviations*: *BMI* body mass index, *SMI* skeletal muscle mass index, *GLFS-25* 25-question Geriatric Locomotive Function Scale, *ODI* Oswestry Disability Index, *PCS* physical component summary, *MCS* mental component summary, *SIA* spinal inclination angle, *TKA* thoracic kyphosis angle, *LLA* lumbar lordosis angle, *TUG* timed up-and-go test, *OLS* one-leg standing time

## Discussion

In this study, spinal sagittal malalignment was related to LS, LBP-related QOL, and physical function, including TUG. Spinal sagittal malalignment showed forward inclination with the progression of LS. In addition, an SIA of ≥ 5° was determined as the cutoff value to predict worse LBP-related QOL status and LS stage 2 and further.

Spinal sagittal malalignment with lumbar kyphosis has been recognized as a cause of chronic LBP, which is caused by fatty degeneration and chronic compartment syndrome of the lumbar back muscles [22, 23]. Sagittal spinopelvic parameters and their correlation with HRQOL outcomes using ODI and Scoliosis Research Society Outcome Instrument have recently received considerable attention [1, 2]. In addition, global sagittal malalignment was reported to affect physical function, such as trunk balance and gait performance [7, 24], which increased the risk of falls and reduced healthy life expectancy. In the present study, SIA was associated with impairments in LBP-related QOL assessed by ODI% and physical functions assessed by TUG, compatible with previous studies. Therefore, the impact of ASD on LBP and HRQOL warrants the same research and health policy attention as other important chronic diseases.

Although sarcopenia and LS have different disease pathophysiologies, both lead to musculoskeletal disorders, reduced mobility function, and hindering ADL. They are partially dependent on each other and directly related to the medical welfare issues of the elderly. Yoshimura et al. reported the prevalence and coexistence of LS, and sarcopenia in a prospective cohort study [25], wherein most participants with sarcopenia coexisted LS. Therefore, LS may play an important role as a trigger in musculoskeletal dysfunction than sarcopenia.

Several reports have shown that sarcopenia is related to various spinal disorders, such as degenerative scoliosis, dropped head syndrome [26], degenerative scoliosis [27], and spinal deformity [28] among selected outpatients in a clinic. However, limited data exist on the relationship between global sagittal alignment and sarcopenia among community-dwelling middle aged and older people. Regarding muscular factors related to sarcopenia, back extensor muscle strength [22] and trunk muscle volume [29–31] were related to a progression of thoracolumbar/lumbar scoliosis and spinal sagittal malalignment, indicating that the trunk muscle plays an important role in the maintenance of physiological spinal alignment. Interestingly, SIA showed the tendency of a relationship with GS, which is recognized as a first-line screening test for sarcopenia (men < 28 kg, women < 18 kg in the Asian Working Group for Sarcopenia [18]) and can be a simple test to predict individual skeletal muscle strength in middle aged and older people with sagittal malalignment. In the present study, SMI was not associated with sagittal malalignment, but trunk muscle mass was tended to be associated with SIA. Therefore, further research on the impact of trunk muscle conditions on spinal deformity or intervention, such as resistance exercise, is needed. Since the number of participants with sarcopenia was small in this study, further research is needed to clarify the impact of sarcopenia on spinal deformity.

Regarding the relationship between spinal sagittal alignment and LS, Muramoto et al. investigated the influence of spinal sagittal alignment on LS in 125 community-living women between 40 and 88 years. Regarding the definition of LS, the participants were divided into two groups: the LS group (corresponding to LS stages 2 and 3, *n* = 26) with a score of ≥ 16 points on the GLFS-25, and the non-LS group (corresponding to LS stages 0 and 1, *n* = 99) with a score of ≤ 15 points. They reported that the SIA was the most relevant spinal parameter for LS, with a cutoff angle of 6° [7]. In the present study, we found that LS diagnosed with the GLFS-25 score was associated with increased SIA assessed by a computer-assisted device and impairments in LBP-related QOL assessed by the ODI%. Unlike the loco-check, the GLFS-25 is also useful for categorizing the three stages of LS. As a result, we showed that SIA was related to the three stages of LS, showing worsened sagittal alignment with the progression of LS. The ROC analysis identified an SIA of 5° as the cutoff value corresponding to LS ≥ stage 2. Although the cutoff value is similar to the results of a previous study reported by Muramoto et al. [7], our study had an approximately three times larger sample size and was the first to show the relationship between sagittal alignment and the newly categorized stages of LS. Since LBP associated with spinal deformity imposes economic and public health burdens in the aging society, we identified an SIA of 5° as the threshold value of the ODI ≥ 20%, corresponding to moderate to severe disability [21]. Since the cutoff values corresponding to impairment of LS and LBP-related QOL coincided excellently, an SIA of 5° is considered one of the indicators to predict HRQOL status or physical performance related to spinal disorders in middle aged and older women.

This study had several limitations. First, subject-selection bias was present since the participants who had participated in the aquatic exercise may have been more active than most average middle aged and older people. Therefore, further studies targeting middle aged and older people with various ADLs should be performed. Second, this study did not consider other musculoskeletal diseases, such as osteoporosis and osteoarthrosis of the lower extremities. Third, the diagnoses of sarcopenia and LS did not strictly follow the recommended diagnostic process. Therefore, further studies which comprehensively assessed musculoskeletal disorders and physical function tests should be performed. Finally, this was a cross-sectional study; therefore, it did not clarify the causal relationship between the spinal alignment and investigating parameters. Longitudinal and interventional studies are required to evaluate this more adequately. Nevertheless, this study is the first to evaluate the relationship between sagittal malalignment severity and the newly categorized stages of LS and indicates that the various stages of LS can be useful to assess physical function and LBP-related QOL in middle aged and older individuals in an anticipated super-aging society.

## Conclusions

LBP-related QOL and physical performance are significantly associated with global sagittal alignment. In particular, global sagittal alignment is closely correlated with the three-stage category of LS. A spinal inclination of 5° is a threshold value to predict exacerbation of LS and LBP-related QOL.

## Data Availability

The datasets used and/or analysed during the current study are available from the corresponding author on reasonable request.

## Abbreviations

ADL: Activities of daily living
ASD: Adult spinal deformity
AWGS: Asian Working Group for Sarcopenia
BMI: Body mass index
GLFS-25: 25-Question geriatric locomotive function scale
GS: Grip strength
HRQOL: Health-related quality of life
JOA: Japanese Orthopaedic Association
LBP: Low back pain
LLA: Lumbar lordosis angle
LS: Locomotive syndrome
MCS: Mental component summary
ODI: Oswestry Disability Index
OLS: One-leg standing time with eyes open
PCS: Physical component summary
ROC: Receiver-operating characteristic
SF-36v2: Short-Form 36 questionnaire version 2
SF-8: Short-Form 8 questionnaire
SIA: Spinal inclination angle
SMI: Skeletal muscle mass index
TKA: Thoracic kyphosis angle
TUG: Timed up-and-go test
